# Genome-Wide Identification and Transcriptome-Based Expression Profiling of the *Sox* Gene Family in the Nile Tilapia (*Oreochromis niloticus*)

**DOI:** 10.3390/ijms17030270

**Published:** 2016-02-23

**Authors:** Ling Wei, Chao Yang, Wenjing Tao, Deshou Wang

**Affiliations:** 1 Key Laboratory of Freshwater Fish Reproduction and Development (Ministry of Education), Key Laboratory of Aquatic Science of Chongqing, School of Life Science, Southwest University, Chongqing 400715, China; weilchdj@swu.edu.cn (L.W.); yangchao_m12@163.com (C.Y.); enderwin@163.com (W.T.); 2 Key Laboratory of Eco-environments in Three Gorges Reservoir Region (Ministry of Education), Chongqing Key Laboratory of Plant Ecology and Resources Research in Three Gorges Reservoir Region, School of Life Science, Southwest University, Chongqing 400715, China

**Keywords:** Nile tilapia, *Sox* gene, genomic structure, transcriptome, gene expression

## Abstract

The *Sox* transcription factor family is characterized with the presence of a Sry-related high-mobility group (HMG) box and plays important roles in various biological processes in animals, including sex determination and differentiation, and the development of multiple organs. In this study, 27 *Sox* genes were identified in the genome of the Nile tilapia (*Oreochromis niloticus*), and were classified into seven groups. The members of each group of the tilapia *Sox* genes exhibited a relatively conserved exon-intron structure. Comparative analysis showed that the *Sox* gene family has undergone an expansion in tilapia and other teleost fishes following their whole genome duplication, and group K only exists in teleosts. Transcriptome-based analysis demonstrated that most of the tilapia *Sox* genes presented stage-specific and/or sex-dimorphic expressions during gonadal development, and six of the group B *Sox* genes were specifically expressed in the adult brain. Our results provide a better understanding of gene structure and spatio-temporal expression of the *Sox* gene family in tilapia, and will be useful for further deciphering the roles of the *Sox* genes during sex determination and gonadal development in teleosts.

## 1. Introduction

*Sox* transcriptional factors are characterized as Sry-related high-mobility group (HMG) box proteins in metazoans. With the availability of whole genome sequence, genome-wide characterization of *Sox* genes has been performed in several animals [1,2,3,4,5], and in total more than 40 members of the *Sox* family have been identified. Based on the sequences of both DNA and proteins, *Sox* gene family is currently divided into 11 groups from A to K [2,5,6]. To date, *Sox* genes have been reported to be involved in not only sex determination and differentiation [7,8,9,10,11], but also the formation of multiple organs, including neuronal system [12,13,14,15], gonad [16,17,18], eye [19,20], pancreas [21,22,23], and cartilage [14,24,25].

Previous reports revealed that the numbers of *Sox* genes greatly varied in animals, namely five in the nematode (*Caenorhabditis elegans*) [26], seven in the calcareous sponge (*Sycon ciliatum*) [1], seven in the sea lamprey (*Petromyzon marinus*) [4], seven in the sea squirt (*Ciona intestinalis*) [27,28], eight in the fruit fly (*Drosophila melanogaster*) [3], nine in the silkworm (*Bombyx mori*) [29], 11 in the sea urchin (*Strongylocentrotus purpuratus*) [30], 14 in the cnidarians (*Nematostella vectensis*) [31], and 20 in the mouse (*Mus musculus*) and human (*Homo sapiens*) [32]. In teleosts, it has been reported that there are 19 in the medaka (*Oryzias latipe*) [2], and 24 *Sox* genes in the pufferfish (*Fugu rubripes*) [5]. Recently, new versions of genome sequences of the Nile tilapia (*Oreochromis niloticus*), zebrafish (*Danio rerio*), and common carp (*Cyprinus carpio*) have been published [33,34,35]. A genome-wide comparative analysis of the *Sox* gene family between the tilapia and other animals including other teleost fishes will be helpful for deciphering the evolutionary process of this gene family.

Previous studies have investigated the potential roles of *Sox* genes in the growth and development of the teleost fishes. For example, several members of the medaka *Sox* family exhibit differential expressions during embryonic development and may play a variety of roles in embryogenesis [2]. Importantly, the medaka *Sox9b* has been shown to be indispensible for the proper proliferation and survival of germ cells in gonads [36]. In addition, evidence from the zebrafish suggests that *Sox7* and *Sox18* play redundant roles in both arteriovenous specification and vascular development [37,38], and *Sox21a* functions as a transcriptional repressor in dorso-ventral patterning during embryonic development [39]. Moreover, only three *Sox* genes, namely, *Sox2*, *Sox14*, and *Sox30*, have been studied in the tilapia [40,41], and *Sox30* has been confirmed to be specifically expressed in gonads [41]. Recently, the transcripomes of multiple adult tissues and different stages of gonadal development in the tilapia have been examined via RNA-Seq method [33,42]. This enables us to carry out transcriptome-based expression profiling of the tilapia *Sox* genes and to obtain more functional evidence for the *Sox* genes in teleosts.

In this study, based on the genome sequence and transcriptome data of the tilapia and other animals, we performed a genome-wide identification and evolutionary analysis of the tilapia *Sox* gene family, and further profiled their spatio-temporal expressions. Our goal is to provide new insight into the evolution and functions of the *Sox* genes in teleosts.

## 2. Results

### 2.1. Identification of the Sox Genes in the Tilapia Genome

We used the amino acids sequence of conserved HMG-box domain of *Sox* transcription factors as query to search against the tilapia genome by a basic local alignment search tool (BLAST). As a result, a total of 27 *Sox* genes, including three previously identified *Sox* genes, namely *Sox2*, *Sox14*, and *Sox30*, were identified in the tilapia genome (Table 1). All the tilapia *Sox* genes could be classified into seven subfamilies, namely, eight members in group B (including five in B1 subgroup and three in B2 subgroup), four in group C, four in group D, six in group E, three in group F, one in group H, and one in group K (Table 1). Interestingly, each of the eight members of the ancestral vertebrate *Sox* genes, namely, *Sox1*, *Sox4*, *Sox6*, *Sox8*, *Sox9*, *Sox10*, *Sox11*, and *Sox14*, has two copies in the tilapia genome, indicating that these *Sox* genes experienced a duplication during the evolution of the tilapia.

### 2.2. Genomic Structure of the Tilapia Sox Genes

**Figure 1:**
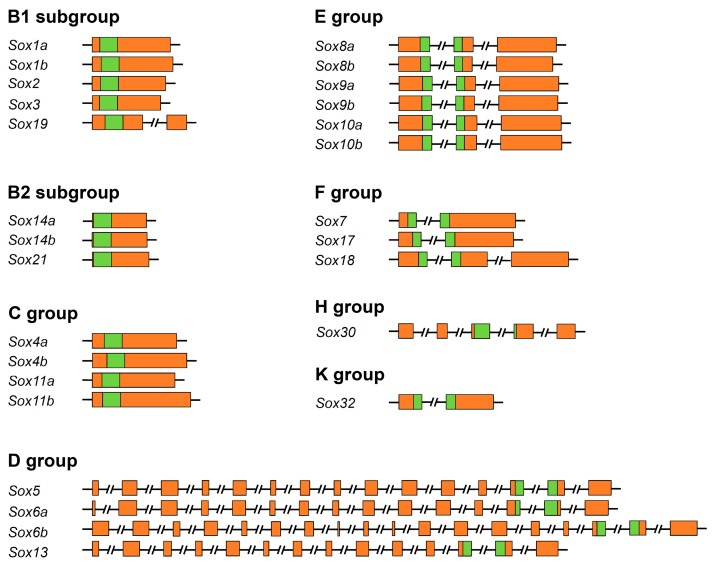
Exon–intron structure of the tilapia *Sox* genes. Rectangle and line with double slash indicate exon and intron, respectively. The HMG-box domain regions and the rest regions of the exons are highlighted with green and brown, respectively.

The exon–intron structure of the tilapia *Sox* genes was further characterized. The results showed that the numbers of intron in each *Sox* gene varied from zero to 17 (Figure 1 and Table 1). No intron was found in 11 of the tilapia *Sox* genes, namely, *Sox1a*, *Sox1b*, *Sox2*, *Sox3*, *Sox4a*, *Sox4b*, *Sox11a*, *Sox11b*, *Sox14a*, *Sox14b*, and *Sox21*. Interestingly, we noted that the *Sox* genes from the same subfamily generally contained similar, even same intron number (Figure 1). For example, all *Sox* genes in group B (including subgroups B1 and B2) had no intron, except for *Sox19*. Two introns were found in *Sox* genes of the group E. More than 14 introns were present in all *Sox* genes that belong to group D. Notably, the HMG boxes in the *Sox* genes from groups D, E, F, H, and K contained only one intron.

**Figure 2:**
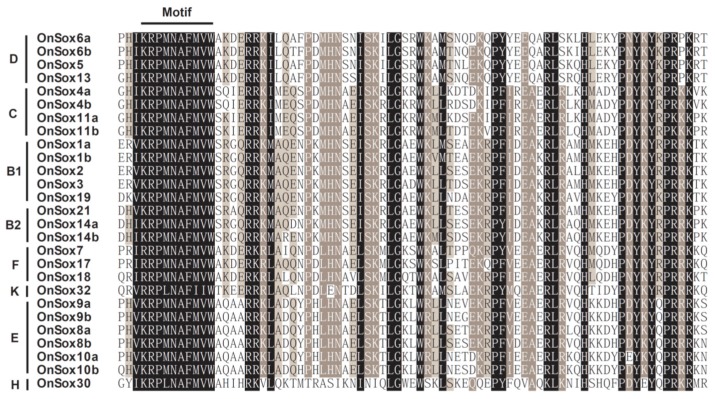
Multiple alignment of HMG-box domain sequences of the tilapia *Sox* proteins. The HMG-box domain of each *Sox* protein was predicted online using SMART program (http://smart.embl-heidelberg.de). ClustalX program was used to carry out a multiple alignment of amino acid sequences of the HMG-box domain of all the tilapia *Sox* proteins.

The amino acid sequences of the HMG boxes of the tilapia *Sox* proteins were aligned. As shown in Figure 2, the core motif of RPMNAFMVW (in the position of 5–13) in the HMG boxes of the tilapia *Sox* proteins, which is responsible for recognizing and binding *cis*-regulatory elements in the promoter of their target genes, is highly conserved. Especially, these motifs are the same among the tilapia *Sox* proteins, except for *Sox30* and *Sox32*.

### 2.3. Comparison of the Sox Genes Among the Tilapia and Other Animals

Given that the tilapia has undergone three rounds of whole genome duplication [43], and the whole genome duplication (WGD) can drive the expansion of gene families [44], we surveyed the number changes of the *Sox* gene members among the tilapia and other analyzed animals with different rounds of genome duplication, from first round (1R) to fourth round (4R). For a comprehensive comparison, we newly identified 49 *Sox* genes in the common carp genome, and updated the number of the *Sox* genes as 27 in the zebrafish (including four newly identified *Sox* genes, namely *Sox12*, *Sox13*, *Sox14a*, and *Sox14b*), 25 in the pufferfish (*Sox32* was newly identified in this study), and 10 in the Florida lancelet (*Branchiostoma floridae*) (Table 2 and Table S1). Group K and group G only existed in teleost fishes and human, respectively. These results, together with the previous reports on the genome-wide identification of the *Sox* genes in other analyzed animals (Table 2 and Table S1), revealed that the number of the *Sox* genes have undergone an expansion following genome duplication in the teleost fishes, and this expansion of the *Sox* gene family mainly occurred in the groups of B, C, E, and K.

**Figure 3:**
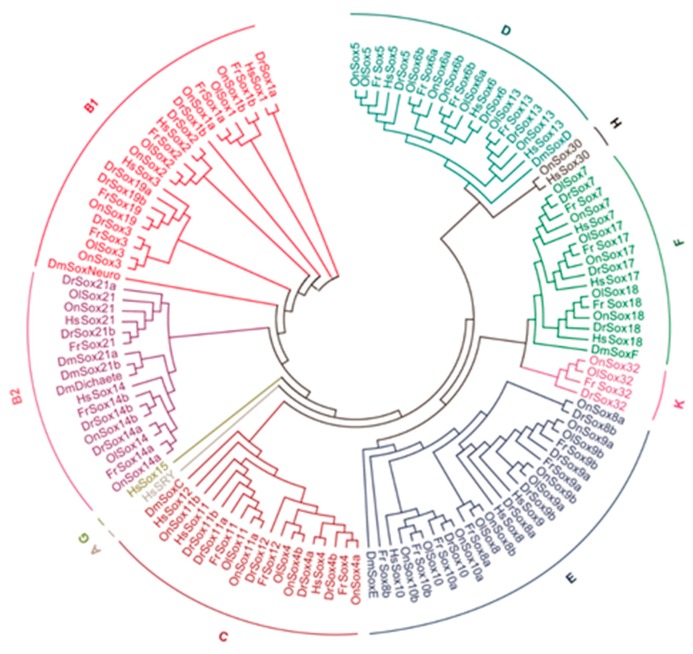
Phylogenetic tree of the *Sox* proteins from the tilapia and the other animals. The amino acid sequences of the HMG-box domain of the *Sox* proteins were used to build a neighbor-joining phylogenetic tree of the *Sox* genes by using MEGA 6.0 program. The sources of the sequences were described in the Materials and Methods Section. On: *Oreochromis niloticus*; Ol: *Oryzias latipes*; Dr: *Danio rerio*; Fr: *Fugu rubripes*; Hs: *Homo sapiens*; Dm: *Drosophila melanogaster*.

We further used amino acid sequences of the HMG-boxes of the *Sox* proteins to build phylogenetic tree of the *Sox* genes among the tilapia and other five selected animals, including zebrafish, pufferfish, medaka, human, and fruit fly. The result showed that all *Sox* genes were grouped into nine groups, including A, B (B1 and B2), C, D, E, F, G, H, and K (Figure 3 and Supplementary Figure S1). Notably, although group K is very close to group F, given that the *Sox32* gene from group K only existed in teleosts and previous report has assigned the *Sox32* gene of the teleost medaka to group K [2], we thus considered all *Sox32* genes from teleosts as group K. Intriguingly, the phylogenetic tree, together with the number variation, revealed that several ancestral *Sox* genes have undergone duplication to form two copies in teleost fishes, including eight members in the tilapia (*i.e.*, *Sox1*, *Sox4*, *Sox6*, *Sox8*, *Sox9*, *Sox10*, *Sox11*, and *Sox14*), six in the zebrafish (*i.e.*, *Sox1*, *Sox4*, *Sox9*, *Sox11*, *Sox19*, and *Sox21*), six in the pufferfish (including *Sox1*, *Sox6*, *Sox8*, *Sox9*, *Sox10*, and *Sox14*), and two in the medaka (*i.e.*, *Sox6* and *Sox9*). The duplication of *Sox9* gene occurred in all teleost fishes. In addition, one duplicate of each ancestral *Sox* gene in the tilapia firstly grouped well together with its orthologs in other fishes, then with the groups containing another duplicate. This indicates that the duplication of these *Sox* genes may have occurred prior to the radiation of teleosts but after the separation of the teleosts from other vertebrates.

### 2.4. Spatial Expression of the Tilapia Sox Genes

**Figure 4:**
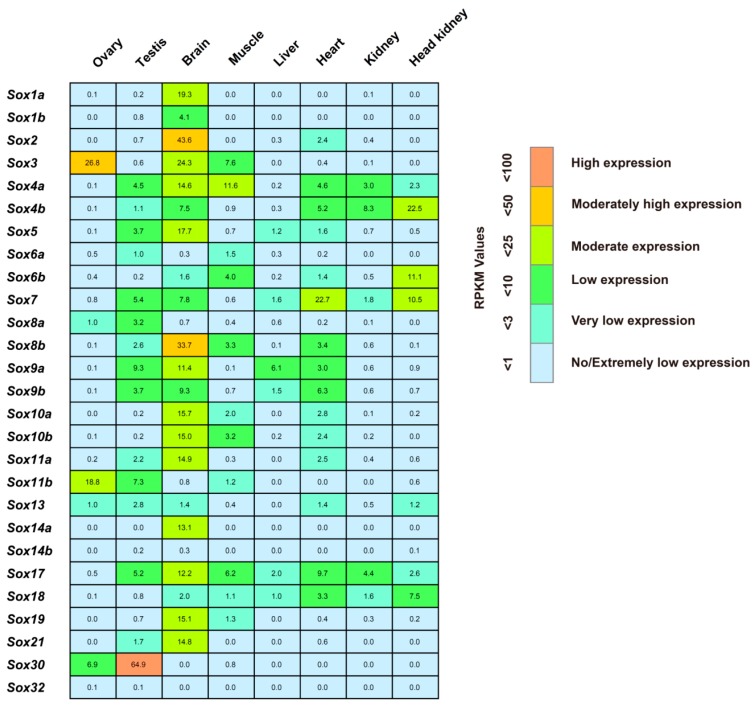
Spatial expression profiles of the tilapia *Sox* genes. The transcriptomes (NCBI accession number: PRJNA78915 and SRR1916191) of multiple tissues of the adult tilapia were used to profile spatial expression of the tilapia *Sox* genes. Numbers within the box indicate the RPKM values.

We next profiled the spatial expression of the tilapia *Sox* genes by using transcriptome data for eight adult tissues of the tilapia, including ovary, testis, brain, muscle, liver, heart, kidney, and head kidney. According to the criteria that a gene is regarded to be expressed if it exhibits an expression level with RPKM (reads per kb per million) value ≥ 1, we found that except for *Sox14b* and *Sox32*, the other 25 *Sox* genes were expressed in at least one of the adult tilapia tissues (Figure 4). The number of the *Sox* genes that were expressed in brain is the largest, reaching to 21. In addition, we observed the expressions of 15 *Sox* genes in testis, 15 in heart, 11 in muscle, seven in head kidney, six in liver, five in ovary, and five in kidney.

Notably, four *Sox* genes showed high expression with RPKM value ≥25, including *Sox3* in ovary, and *Sox30* in testis, and *Sox2* and *Sox8b* in brain (Figure 4). Moreover, 16 *Sox* genes were moderately expressed in different adult tissues, showing RPKM value that were greater than 10 and less than 25, including *Sox11b* in ovary, *Sox1a*, *Sox3*, *Sox4a*, *Sox5*, *Sox9a*, *Sox10a*, *Sox10b*, *Sox11a*, *Sox14a*, *Sox17*, *Sox19*, and *Sox21* in brain, *Sox4a* in muscle, *Sox7* in heart, and *Sox4b*, *Sox6b*, and *Sox7* in head kidney. Interestingly, several *Sox* genes showed a tissue-specific expression, including *Sox8a* in ovary, and *Sox1a*, *Sox1b, Sox2, Sox14a*, *Sox19*, and *Sox21* in brain.

### 2.5. Temporal Expression of the Sox Genes in the Tilapia Gonads

**Figure 5:**
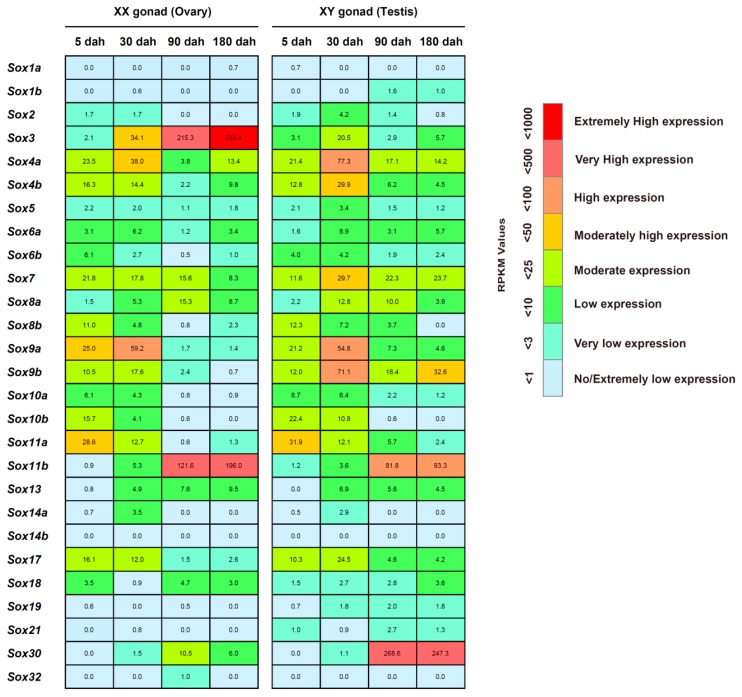
Temporal expression profiles of the *Sox* genes in the XX and XY gonads of the tilapia. The transcriptomes (NCBI accession number: SRA055700) of the tilapia gonads in four developmental stages comprising of 5, 30, 90 and 180 dah were used to analyze the expression profiles of the *Sox* genes during gonadal development. Numbers within the box indicate the RPKM values.

We used transcriptome data of the tilapia XX (ovary) and XY (testis) gonads at four developmental stages, namely 5, 30, 90 and 180 days after hatching (dah), to profile the temporal expression of the tilapia *Sox* genes. The results revealed that except for *Sox1a*, the other 26 *Sox* genes were expressed in XX (ovary) and/or XY (testis) gonads in at least one development stage (Figure 5). Among these expressed *Sox* genes, nine members (*i.e.*, *Sox3*, *Sox4a*, *Sox4b*, *Sox7*, *Sox9a*, *Sox9b*, *Sox11a*, *Sox11b*, and *Sox30*) and four members (*i.e.*, *Sox8a*, *Sox8b*, *Sox10b*, and *Sox17*) presented high expression and a moderate expression in XX (ovary) and/or XY (testis) gonads in at least one developmental stage, respectively.

We further characterized the expression change of each *Sox* gene during gonadal development of the tilapia. As shown in Figure 5, the expressions of several *Sox* genes were gradually elevated during gonadal development, such as *Sox3* and *Sox11b* in XX gonads (ovary) as well as *Sox11b* and *Sox30* in XY gonads (testis), which showed great elevation in the late two stages of 90 dah and 180 dah. Moreover, in XX (ovary) and/or XY (testis) gonads, nine *Sox* genes, namely *Sox4a*, *Sox4b*, *Sox8b*, *Sox9a*, *Sox9b*, *Sox10a,* *Sox10b*, *Sox11a*, and *Sox17*, exhibited high expression in the early two stages of 5 dah and 30 dah, and of which the expressions of two *Sox* genes (*Sox10b* and *Sox11a*) and four *Sox* genes (*i.e.*, *Sox4a*, *Sox4b*, *Sox9a*, and *Sox9b*) were very highly enriched at 5 dah and 30 dah, respectively.

### 2.6. Sexually Dimorphic Expression of the Sox Genes in the Tilapia Gonads

We examined whether the tilapia *Sox* genes exhibited sexually dimorphic expressions in gonads. First, a paired comparative analysis demonstrated that several *Sox* genes were specifically expressed at a time point during the development of the tilapia gonads (Figure 5), such as *Sox1b* and *Sox2* in XY gonads (testis) at both 90 dah and 180 dah, and *Sox8b at* 180 dah and *Sox32* at 90 dah in XX gonads (ovary). But, the expression level of these tilapia *Sox* genes was very low.

**Figure 6:**
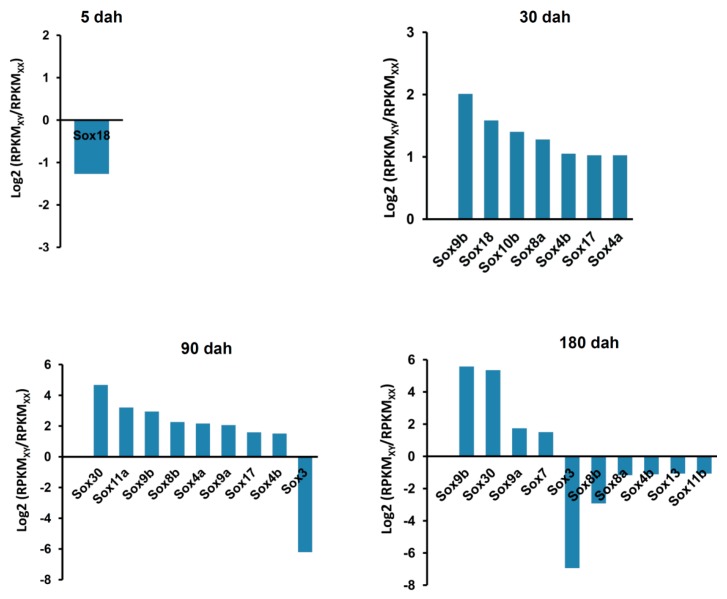
Differential expressions of the *Sox* genes between XX and XY gonads of the tilapia at different developmental stages. The ratio of RPKM value for the expression of each *Sox* gene in XX (ovary) and XY (testis) gonads in each developmental stage was calculated. If the log2 of the ratio is ≥1 or ≤−1, this *Sox* gene was considered as sexual dimorphically expressed *Sox* genes in a developmental stage.

Second, we observed there were 1, 7, 10, and 10 *Sox* genes to display sexually dimorphic expression at 5 dah, 30 dah, 90 dah, and 180 dah, respectively (Figure 5 and Figure 6). In detail, only *Sox18* was up-regulated in XX gonads (ovary) at 5 dah. At 30 dah, seven *Sox* genes, containing *Sox4a*, *Sox4b*, *Sox8a*, *Sox9b*, *Sox10b*, *Sox17*, and *Sox18*, were up-regulated in XY gonads (testis)*.* In addition, we noted that eight *Sox* genes (*i.e.*, *Sox4a*, *Sox4b*, *Sox8b*, *Sox9a*, *Sox9b*, *Sox11a*, *Sox17*, and *Sox30*) and four *Sox* genes (*i.e.*, *Sox7*, *Sox9a*, *Sox9b*, and *Sox30*) were up-regulated in XY gonads (testis) at 90 and 180 dah, respectively. Conversely, several *Sox* genes showed up-regulation in XX gonads (ovary), including *Sox3* at 90 dah and 6 *Sox* genes (*i.e.*, *Sox3*, *Sox4b*, *Sox8a*, *Sox8b*, *Sox11b*, and *Sox13*) at 180 dah. Intriguingly, although *Sox9a* was highly expressed in both XX (ovary) and XY (testis) gonads at 5 and 30 dah (Figure 5 and Figure 6), it began to be differentially expressed in gonads, showing an up-regulation in XY gonads (testis) at 90 and 180 dah. Besides, *Sox9b* expression exhibited a significant up-regulation in XY gonads (testis) at 30, 90 and 180 dah. Consistently, quantitative RT-PCR examination revealed similar developmental and sexually dimorphic expressions of *Sox9a* and *Sox9b* in the tilapia gonads, and further confirmed that compared to XY gonads (testis), the expression of the *Sox9a* gene displayed a significant up-regulation in XX gonads (ovary) at 5 and 10 dah.

## 3. Discussion

*Sox* transcription factor family is exclusively discovered in animals to date and contributes to modulate various biological processes, like sex determination and gonadal development [45]. Recently, genome-wide characterization of the *Sox* gene family has been extensively performed in the metazoan, such as the nematode, insect, mammal and teleost. In this study, based on the recently published genome sequences for two lineages of teleost fishes, the tilapia and zebrafish [33,34], we identified 27 *Sox* genes in the tilapia and 27 *Sox* genes in the zebrafish.

Comparative analysis revealed several evolutionary perspectives of the *Sox* gene family during the separation of fish species from other animals. First, the *Sox* genes have undergone a continuous expansion in the teleost fishes following their whole genome duplication, which is supported by our finding shown in Table 2 and previous finding [46]. Intriguingly, although the orthologs of several mammalian *Sox* genes in the teleost fishes with 3R whole genome duplication have undergone duplication to generate two copies, different duplicates from an ancestral *Sox* gene have been demonstrated to exhibit splitting roles, like *Sox9*, *Sox11*, and *Sox21* in the zebrafish [25,47,48]. Second, several *Sox* genes are exclusively present in vertebrates. For instance, the group K member *Sox32* gene was specifically identified in teleosts. Previous study demonstrated that *Sox32* is essential for the endodermal differentiation in the zebrafish [49], suggesting that it may be evolved to control the formation of specific organs in teleosts. In addition, the homolog of the human and chicken *Sox30* gene was also discovered in the tilapia genome, consistent with our previous observation [41], but absent in other four teleosts, namely the zebrafish, pufferfish, medaka, and common carp. Curiously, *Sox30* could be found in the teleosts guppy and channel catfish [41]. Undoubtedly, the evolution and functions of *Sox30* in fishes and other vertebrates are somewhat complex and are worthy to be further investigated.

*Sox* transcription factors are involved in diverse physiological processes in animals through transcriptional activation and/or repression of their target genes in tissue- or development-specific manners [45,50]. It is very interesting that six *Sox* genes that belong to group B of the *Sox* family, including *Sox1a*, *Sox1b, Sox2, Sox14a*, *Sox19*, and *Sox21*, exhibited a specific expression in brain of the adult tilapia (Figure 5). Notably, four members of the *SoxB1* subfamily, *Sox1a*, *Sox1b, Sox2,* and *Sox19*, have been characterized as the markers of the neural progenitor and stem cells throughout the vertebrate central nervous system (CNS) including brain, and contribute to not only regulating pluripotency but also mediate self-renewal and differentiation of neural progenitor and stem cells [51]. *Sox21* from the *SoxB2* subfamily functions as a counteracting partner of the *SoxB1* genes to regulate neuron differentiation and promotes neurogenesis in vertebrate [52,53]. Accordingly, we proposed that the specific expression of the group B *Sox* genes in the adult brain may be necessary for the neurogenesis or the maintenance of specific biological processes in the tilapia brain.

Generally, several key biological events occur at these different time points during gonadal development of the tilapia, like sex determination and differentiation around 5–10 dah, the initiation of germ cell meiosis and oogenesis in the XX gonads (ovary) at 30 dah, the initiation of spermatogenesis in the XY gonads (testis) at 90 dah, and sperm maturation in the XY gonads (testis) and vitellogenesis in the XX gonads (ovary) at 180 dah [42,54]. A striking finding of our study is that several *Sox* genes exhibited a stage-specific and/or sexually dimorphic expression in the tilapia gonads, which provides new insights into their potential roles in gonadal development of the tilapia.

Our results revealed that *Sox3* and *Sox30* were very highly expressed in XX (ovary) and XY (testis) gonads during 90–180 dah, respectively (Figure 5 and Figure 6), indicating that these two genes may be required for oogenesis and spermatogenesis but not for sex determination. In fact, previous reports have demonstrated that the homolog of the tilapia *Sox3* gene is required for gonadal function in the mouse and for oogenesis in the protogynous hermaphrodite fish (*Halichoeres poecilopterus*) [55,56], and the mice *Sox30* gene is highly expressed in testis and regulates spermatogonial differentiation and spermatogenesis during testis development [57].

**Figure 7:**
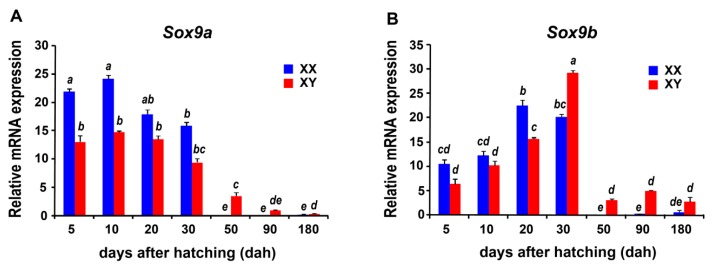
Quantitative RT-PCR examination of the *Sox9a* (**A**) and *Sox9b* (**B**) expressions in the XX and XY gonads of the tilapia. The quantitative RT-PCR experiment was performed in triplicates using pooled monosex fish cDNAs in each of the selected developmental stages. Values represent the relative mRNA expression in relation to the internal control (*β-actin* gene). Data were expressed as the mean ± SD of the triplicates. Column error bars with the same letter are not significantly different at *p* < 0.05 as determined using Least Significant Difference (LSD) test.

We also noted, as shown in Figure 5, Figure 6 and Figure 7, both *Sox9a* and *Sox9b* were highly expressed in gonads before 30 dah, a period that the completion of sex determination and the initiation of sex differentiation occur [54,58], indicating they may be involved in these processes during gonadal development of the tilapia. However, *Sox9a* expression was significantly higher in XX gonads (ovary) than that in XY gonads (testis) at 5 and 10 dah, whereas *Sox9b* expression was significantly higher in XY gonads (testis) than that in XX gonads (ovary) at 30 dah, indicating *Sox9a* may be mainly involved in the regulation of sex determination and ovarian differentiation, and *Sox9b* may regulate testicular differentiation in the tilapia.

In addition, *Sox17* was confirmed to be highly expressed in both XX (ovary) and XY (testis) gonads of the tilapia at 5 dah (sex determination and differentiation) and 30 dah (initiation of germ cell meiosis and oogenesis in the XX gonads (ovary). Given that previous observation in mouse that *Sox17* mediates the specification of parietal endoderm cells during embryogenesis [59,60], the early expression of the tilapia *Sox17* suggests that it might also be involved in the differentiation and specification of the tilapia gonads. Interestingly, *Sox11a* and *Sox11b* were highly expressed at 5–30 and 90–180 dah (the initiation of spermatogenesis in the XY gonads (testis) at 90 dah, and sperm maturation in the XY gonads (testis) as well as vitellogenesis in the XX gonads (ovary) at 180 dah), respectively. This suggests that *Sox11a* may regulate gonadal differentiation while *Sox11b* may be involved in spermatogenesis and vitellogenesis in the tilapia. Undoubtedly, the real roles of these *Sox* genes in the development of the tilapia gonads need to be clarified in the future studies.

## 4. Materials and Methods

### 4.1. Animal Rearing

The Nile tilapia fishes used in this study were reared in large tanks with recirculating freshwater at ambient temperature (26 °C) and under natural photoperiod. All females (XX) and males (XY) progenies were obtained by crossing the normal female (XX) with the sex-reversed XX pseudomale and YY supermale, respectively [61]. All animal experiments were performed following the regulations of the Guide for Care and Use of Laboratory Animals at Southwest University, Chongqing, China.

### 4.2. Genome-Wide Identification of the Sox Genes

The genome sequences and predicted protein-coding gene sets of the tilapia, zebrafish and common carp were downloaded from the online databases (http://asia.ensembl.org/Oreochromis_niloticus/Info/Index; http://asia.ensembl.org/Danio_rerio/Info/Index; http://www.carpbase.org/download_home.php). To identify candidate *Sox* genes in these three fish species, we first used the protein sequence of conserved HMG box domain (InterPro ID: IPR009071) for *Sox* protein to search against their predicted protein-coding gene sets by using local BLASTP program, with an *E* value threshold of 10^−5^. Secondly, given that the annotation of the zebrafish genes should be more precise, we used the amino acid sequences of each *Sox* gene from the zebrafish to search against the genome assemblies of tilapia and common carp via TBLASTN program with an *E* value threshold of 10^−5^, and the results from this search could be used to check the accuracy of the predicted *Sox* genes from the tilapia and common carp. The identified *Sox* genes were named according to the principle described in the previous report [6]. In addition, the genomic distribution of the *Sox* genes from these three fish species were characterized by mapping the amino acid sequences of each *Sox* gene on the genome assembly by using TBLASTN program.

To perform a comparative analysis, we collected the previously identified *Sox* genes of seven animals in the databases of NCBI (http://www.ncbi.nlm.nih.gov) and Ensembl (http://www.ensembl.org/), including the fruit fly, pufferfish, medaka, human, western clawed frog (*Xenopus tropicalis*), and chicken (*Gallus gallus*), according to the previous reports [2,3,5,32].

### 4.3. Phylogenetic Analysis

The amino acid sequence of the HMG box of all *Sox* proteins from six analyzed species, including the tilapia, zebrafish, fruit fly, pufferfish, medaka, and human were extracted base on the SMART analysis [62]. Multiple alignment of the HMG box of *Sox* proteins was performed using ClustalX program [63]. The neighbor-joining phylogenetic tree of the *Sox* genes were constructed by using MEGA 6.0 program [64], with a bootstrap of 1000 replicates.

### 4.4. Transcriptome-Based Analysis of Expression Profiling of the Sox Genes

The transcriptome data of the developing gonads and adult tissues were used to profile the temporal-spatial expressions of the tilapia *Sox* genes. Our previous study has sequenced the transcriptomes (NCBI accession number: SRA055700) of XX (ovary) and XY (testis) gonads at four different stages of the tilapia development, namely 5, 30, 90 and 180 days after hatching (dah) [42]. In addition, the transcriptomes (NCBI accession number: PRJNA78915 and SRR1916191) of the tilapia adult tissues were generated from brain, muscle, liver, heart, kidney, ovary, testis, and head kidney [33,65].

A normalized measure of RPKM value was used to characterize the expressions of the tilapia *Sox* genes. A threshold of RPKM value ≥ 1 was used to determine a reasonable expression for each *Sox* gene in a specific tissue at a specific time points [66,67]. The method described in our previous report was used to identify *Sox* genes sexually dimorphically expressed in gonads (XX or XY gonads) at each developmental stage [42]. Briefly, at each stage, *Sox* genes that were expressed specifically either in the XX or XY gonad only were classified as XX or XY-specific, whereas among the *Sox* genes expressed in both XX and XY gonads, those meeting the statistical criteria of both “FDR ≤ 10^−2^” and “|log_2_ (XX_RPKM/ XY_RPKM)| ≥ 1 or ≤ −1” were classified as differentially expressed candidates.

### 4.5. Gene Expression Profiling by Quantitative RT-PCR

Quantitative RT-PCR experiment was used to confirm the temporal-spatial expressions of the tilapia *Sox* genes. The gonads from monosex fishes (XX and XY) were dissected at 5, 10, 20, 30, 50, 90, and 180 dah. Different amount of gonads were collected from each sex at each developmental stage as a pooled sample, namely, approximate 50 gonads for each of two early stages (5 and 10 dah), 10 gonads for each of two stages (20 and 30 dah), 5 gonads for 50 dah, and 3 gonads for each of the two late stages (90 and 180 dah). Three samples were prepared for each stage to perform qRT-PCR experiments in triplicate. Total RNA was extracted from each sample, then treated by DNase, and immediately reverse-transcribed into cDNA using M-MLV reverse transcriptase (Invitrogen, Carlsbad, CA, USA). Quantitative RT-PCR examination was carried out according to the protocol of PlatinumSYBR Green qPCR SuperMix UDG kit (Invitrogen). The tilapia *β*-a*ctin* gene (NCBI accession number: EF206796) was used as an internal control. The primers used here were listed in Supplemental Table S2. The primer pair covers exons 1 and 2 and spanned intron 1 of the tilapia *Sox9a* and *Sox9b*. The raw data were analyzed by using one-way ANOVA and a Fisher's Least Significant Difference (LSD) test, and *p* < 0.05 was considered to be significant.

## 5. Conclusions

*Sox* transcription factors play important roles in animal development. In this study, a genome-wide analysis identified the varied numbers of the *Sox* genes in the tilapia (27), zebrafish (27), and common carp (49). Comparative analysis revealed that the *Sox* genes have undergone duplication in teleosts following their whole genome duplication after their separation from the other vertebrate species. Transcriptome-based expression profiling uncovered the tissue-, stage-, or sex-specific expressions of the tilapia *Sox* genes. The exact roles of these differentially expressed *Sox* genes during the tilapia development need to be precisely characterized in the future.

## Figures and Tables

**Table 1:** Inventory of *Sox* genes in the tilapia genome.

Group	Gene	NCBI ID	Linkage Group	Position	Intron Number	Length (aa)	HMG-Box Position
B1	*Sox1a*	XP_005457843.1	16–21	12,271,122–12,353,293	0	344	35–109
	*Sox1b*	XP_005450142.1	unknown	7,601,713–7,604,245	0	354	41–115
	*Sox2*	XP_003457401.1	17	280,838–283,149	0	322	38–112
	*Sox3*	XP_005467513.1	2	13,569,507–13,587,072	0	300	33–107
	*Sox19*	XP_003458966.1	3	282,331–286,532	1	307	57–131
B2	*Sox14a*	XP_005451233.1	23	7,181,057–7,183,304	0	238	6–80
	*Sox14b*	XP_003438076.1	18	5,299,795–5,300,529	0	241	6–80
	*Sox21*	XP_003447333.1	16–21	14,804,010–14,806,286	0	248	6–80
C	*Sox4a*	XP_005450837.1	22	19,810,503–19,814,320	0	371	55–129
	*Sox4b*	XP_005450601.1	11	10,635,379–10,640,355	0	414	65–139
	*Sox11a*	AAR01937.1	19	1,462,641–1,463,732	0	363	44–118
	*Sox11b*	XP_005452537.1	15	7,943,145–7,945,227	0	433	48–122
D	*Sox5*	XP_005451840.1	17	29,053,787–29,297,905	15	773	566–640
	*Sox6a*	XP_003442343.2	1	665,105–766,767	14	778	568–646
	*Sox6b*	XP_005460016.1	7	667,787–732,752	17	838	613–691
	*Sox13*	XP_005450158.1	5	6,796,676–6,846,010	15	664	461–535
E	*Sox8a*	XP_003438722.1	8–24	6,051,795–6,054,972	2	479	96–170
	*Sox8b*	XP_003450163.1	unknown	1,216,205–1,219,881	2	464	98–172
	*Sox9a*	XP_005448042.1	8–24	5,686,065–5,688,370	2	500	105–179
	*Sox9b*	XP_003450167.1	unknown	1,462,602–1,466,515	2	484	102–176
	*Sox10a*	XP_003447925.1	6	19,531,528–19,536,616	2	500	107–181
	*Sox10b*	XP_005468484.1	4	7,506,891–7,515,424	2	503	105–179
F	*Sox7*	XP_005460371.1	15	17,570,516–17,573,991	1	407	41–115
	*Sox17*	XP_003439290.1	9	14,405,231–14,407,376	1	397	63–137
	*Sox18*	XP_003459774.1	unknown	18,060–22,872	2	565	90–164
H	*Sox30*	XP_003447014.1	unknown	3,119,307–3,123,369	4	353	122–196
K	*Sox32*	XP_003439409.1	9	3,127,363–3,128,440	1	310	35–109

**Table 2:** Number variation of *Sox* genes in the Nile tilapia and the other surveyed animals.

Group	Common Carp (4R)	Nile Tilapia (3R)	Zebrafish (3R)	Pufferfish (3R)	Medaka (3R)	Human (2R)	Western Clawed (2R)	Chicken (2R)	Florida Lancelet (1R)	Fruit Fly
A	-	-	-	-	-	1	-	-		-
B1	12	5	6	5	3	3	3	3	3	1
B2	8	3	4	3	2	2	2	2	2	3
C	8	4	5	3	2	3	2	3	1	1
D	3	4	3	4	4	3	3	3	1	1
E	10	6	5	6	4	3	3	3	1	1
F	6	3	3	3	3	3	4	3	1	1
G	-	-	-	-	-	1	-	-	-	-
H	-	1	-	-	-	1	-	1	1	-
I	-	-	-	-	-	-	1	-	-	-
J	-	-	-	-	-	-	-	-	-	-
K	2	1	1	1	1	-	-	-	-	-
Total	49	27	27	25	19	20	18	18	10	8

## Supplementary Materials

Supplementary materials can be found at http://www.mdpi.com/1422-0067/17/3/270/s1.

## Abbreviations

HMG: high-mobility group
1R: first round of genome duplication
4R: fourth round of genome duplication
dah: days after hatching
RPKM: reads per kb per million read
