# Supplementary material for: Genome-Wide Identification and Transcriptome-Based Expression Profiling of the Sox Gene Family in the Nile Tilapia (Oreochromis niloticus)
Source: Int J Mol Sci. 2016 Feb 23;17(3):270. doi: 10.3390/ijms17030270 (PMC4813134; doi:10.3390/ijms17030270)
Supplement: Supplementary file 1 [file ijms-17-00270-s001.zip › ijms-116732-Supplementary Materials/ijms-116732-Supplementary Figure S1.pdf]

Supplementary Materials: Genome-Wide Identification and Transcriptome-Based Expression Profiling of the *Sox* Gene Family in the Nile Tilapia (*Oreochromis niloticus*)

Ling Wei, Chao Yang, Wenjing Tao and Deshou Wang

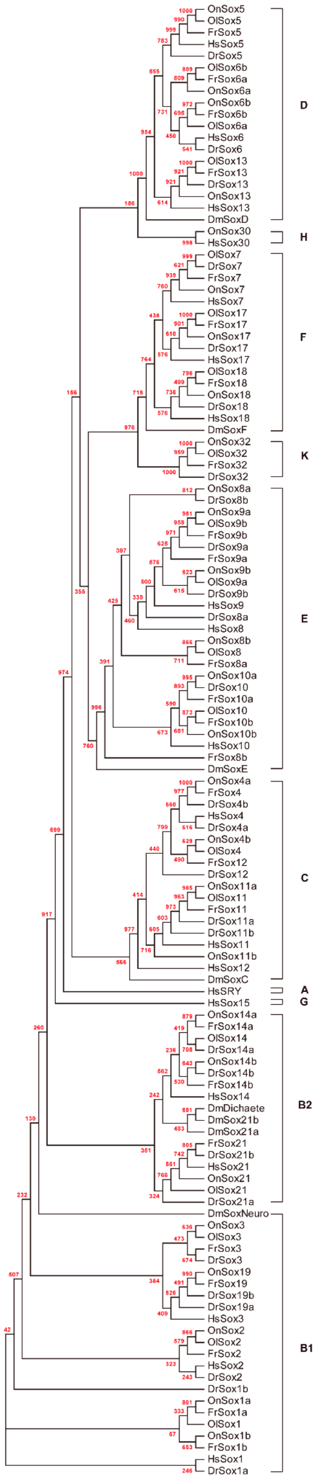

Figure S1. Bootstrap value-containing phylogenetic tree. On, *Oreochromis niloticus*; Ol, *Oryzias latipes*; Dr, *Danio rerio*; Fr, *Fugu rubripes*; Hs, *Homo sapiens*; Dm, *Drosophila melanogaster*.
